# Over-expression of eukaryotic translation initiation factor 4 gamma 1 correlates with tumor progression and poor prognosis in nasopharyngeal carcinoma

**DOI:** 10.1186/1476-4598-9-78

**Published:** 2010-04-16

**Authors:** Luxia Tu, Zhen Liu, Xiufang He, Ying He, Huiling Yang, Qingping Jiang, Siming Xie, Guanghui Xiao, Xin Li, Kaitai Yao, Weiyi Fang

**Affiliations:** 1 Cancer Research Institute, key lab for transcriptomics and proteomics of human fatal diseases supported by ministry of education and Guangdong Province, Southern Medical University, 510515, Guangdong Province, PR China; 2 Department of Otorhinolaryngology, People's hospital of Zhongshan City, 528403, Guangdong Province, PR China; 3 Department of Otorhinolaryngology, Nanfang hospital of Southern Medical University, 510515, Guangdong Province, PR China; 4 Department of Pathology of the first affiliated hospital, Nanchang University, Jiangxi Province, PR China, 330006; 5 Institute of Clinical Medicine, First Affiliated Hospital of University of South China, Hengyang City, 421001, Hunan Province, PR China

## Abstract

**Background:**

The aim of the present study was to analyze the expression of eukaryotic translation initiation factor 4 gamma 1 (*EIF4G1*) in nasopharyngeal carcinoma (NPC) and its correlation with clinicopathologic features, including patients' survival time.

**Methods:**

Using real-time PCR, we detected the expression of *EIF4G1 *in normal nasopharyngeal tissues, immortalized nasopharyngeal epithelial cell lines NP69, NPC tissues and cell lines. *EIF4G1 *protein expression in NPC tissues was examined using immunohistochemistry. Survival analysis was performed using Kaplan-Meier method. The effect of *EIF4G1 *on cell invasion and tumorigenesis were investigated.

**Results:**

The expression levels of *EIF4G1 *mRNA were significantly greater in NPC tissues and cell lines than those in the normal nasopharyngeal tissues and NP69 cells (*P *< 0.001). Immunohistochemical analysis revealed that the expression of *EIF4G1 *protein was higher in NPC tissues than that in the nasopharyngeal tissues (*P *< 0.001). In addition, the levels of *EIF4G1 *protein in tumors were positively correlated with tumor T classification (*P *= 0.039), lymph node involvement (N classification, *P *= 0.008), and the clinical stages (*P *= 0.003) of NPC patients. Patients with higher *EIF4G*1 expression had shorter overall survival time (*P *= 0.019). Multivariate analysis showed that *EIF4G1 *expression was an independent prognostic indicator for the overall survival of NPC patients. Using shRNA to knock down the expression of *EIF4G1 *not only markedly inhibited cell cycle progression, proliferation, migration, invasion, and colony formation, but also dramatically suppressed *in vivo *xenograft tumor growth.

**Conclusion:**

Our data suggest that *EIF4G1 *can serve as a biomarker for the prognosis of NPC patients.

## Introduction

Nasopharyngeal carcinoma (NPC) is a common malignant disease in Southern China where its annual incidence rate is more than 20 cases per 100,000 populations. Men are twice as likely to develop NPC as women. The incidence generally increases from ages 20 to 50 [1]. Because the primary anatomical site of tumor growth is located in a cryptic area and the disease is usually asymptomatic at early stages, the NPC patients tend to present at a more advanced stage with higher metastatic potential. Therefore, it is of great interest to find effective biomarkers for early diagnosis of as well as therapeutic targets for this malignancy.

The tumorigenesis and metastasis of NPC are complex and continuous processes that involve a cascade of oncogene activations [1,2]. Latent membrane protein 1 (*LMP1*), a protein encoded by Epstein-Barr virus (EBV), is considered as a major oncogenic protein promoting the development of NPC. *LMP1*-positive cells have greater mobility, leading to higher metastatic potential [3] and faster disease progression [4]. Protein *XBP-1 *induces *LMP1 *expression, and knockdown of *XBP-1 *blocks up-regulation of *LMP1 *in NPC cells. Furthermore, *XBP-1 *significantly correlates with *LMP1 *expression in NPC tumor biopsies, suggesting that *XBP-1 *can promote virus-associated cancer in a unique way by driving expression of a viral oncogene [5]. *Survivin *is an inhibitor of apoptosis and promoter of cell proliferation, and it is usually absent in normal adult tissues but present in numerous tumors [6]. In NPC, *survivin *has been constantly found to be over-expressed [7]. Inhibiting survivin expression can decrease NPC cell viability as well as increase tumor radiosensitivity [8].

In a previous study, we investigated differentially expressed genes between non-cancerous nasopharyngeal tissues and NPC tissues using cDNA microarray. One of the genes showed markedly increased expression in NPC was eukaryotic translation initiation factor (*EIF4G1*) [7]. *EIF4G1 *participates in protein translation by serving as a scaffold for several other initiation factors [9]. In addition, recent studies have found that EIF4G1 possesses tumorigenesis activities including promoting angiogenesis [10], inducing malignant transformation [11], and inhibiting apoptosis [12]. In a variety of cancers, including NPC, lung cancer, and breast cancer, the expression levels of *EIF4G1 *are significantly up-regulated [13-16].

In the present study, we investigated the role of *EIF4G1 *in the pathogenesis of NPC, and examined its association with clinicopathologic features, including the survival of patients with NPC. We identified the effects of *EIF4G1 *on cell growth, invasion, and *in vivo *tumorigenesis, confirmed the increased expression levels of *EIF4G1 *in primary NPC tissues and cell lines, and found an inverse correlation between the levels of *EIF4G1 *and clinical outcome of patients with NPC.

## Results

### *EIF4G1 *Was Highly Expressed in NPC Tissues and Cell Lines

**Figure 1:**
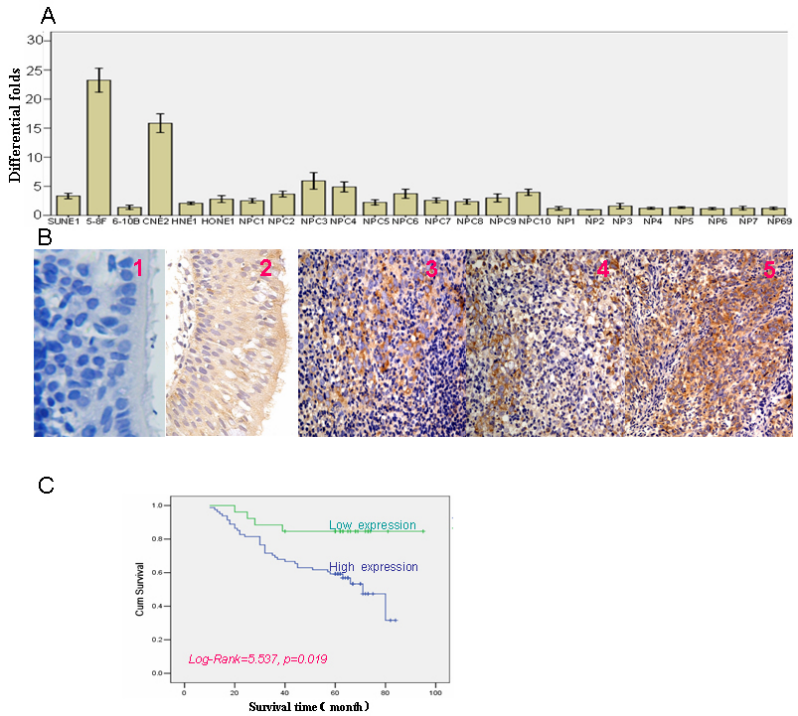
**The increased expression levels of *EIF4G1 *mRNA and protein in NPC tumor tissue and cell lines and their association with the overall survival of NPC patients**. **A**. *EIF4G1 *mRNA expression in NPC cell lines, tumor tissues, and non-cancerous nasopharyngeal (NP) tissues. *EIF4G1 *mRNA level was relatively high in NPC tissue, NPC cell lines (except 6-10B cells) comparing to nasopharyngeal tissue and NP69 cells. **B**. *EIF4G1 *protein expression in NPC and nasopharyngeal tissues.(1) Negative control; (2) Weak expression of *EIF4G1 *in nasopharyngeal tissues; (3-5) Strong expression of *EIF4G1 *in NPC tissues (magnification 400×). **C**. Kaplan--Meier survival analysis of the overall survival time in 107 NPC patients based on the levels of *EIF4G1 *protein expression. The log-rank test was used.

We first set out to confirm our previous observation that *EIF4G1 *was a differentially expressed gene between normal and cancerous nasopharyngeal tissues. Using real-time PCR to measure the expression of *EIF4G1 *transcripts, we found that *EIF4G1 *expression was significantly increased in freshly isolated NPC tissues (n = 10) and NPC cell lines (n = 6) in comparison to freshly isolated nasopharyngeal tissues (n = 7) and the immortalized human nasopharyngeal epithelial cell lines NP69 (Figure 1A) (***P ***< 0.001). Among the 6 NPC cell lines, 5-8F cells had the highest expression levels of *EIF4G1 *(Figure 1A); this cell line is also highly tumorigenic and metastatic [17,18], suggesting of 5-8F cells as a good model system for studying the functions of endogenous *EIF4G1 *by loss-of-function approach.

In addition to the transcript level, using immunohistochemistry, we also found that the levels of *EIF4G1 *protein were significantly greater in formalin-fixed paraffin-embedded (FFPE) NPC tissue samples (n = 137) than the non-cancerous nasopharyngeal tissue samples (n = 53) (*P *< 0.001) (Table 1). We observed the expression *of EIF4G1 *protein in the cytoplasm of non-cancerous and malignant nasopharyngeal epithelial cells (Figure 1B).

**Table 1:** Protein expression of *EIF4G1 *in NPC and NP and Correlation of clinicopathological features of the patients with NPC and expression of *EIF4G1 *protein

	*EIF4G1 *protein expression					
	
Characteristics	N	Negative(n)	Low(n)	Medium(n)	High(n)	P value
Group						
NPC	132	2	27	41	62	
NP	53	4	26	19	4	*p *= 0.000
Sex						
Male	94	2	19	31	42	
Female	38	0	8	10	20	*p *= 0.477
Age						
≥45 years	81	2	15	24	40	
< 45 years	51	0	12	17	22	*p *= 0.557
Smoking						
No	100	0	21	29	50	
Yes	32	2	6	12	12	*p *= 0.226
T classfication						
T1+T2	91	2	22	29	38	
T3+T4	41	0	5	12	24	*p *= 0.039*
N classfication						
N0+N1	57	2	14	22	19	
N2+N3	75	0	13	19	43	*p *= 0.008*
M classfication						
M0	127	2	26	40	59	
M1	5	0	1	1	3	*p *= 0.625
Clinical stage						
I+II		2	11	16	11	
III+IV	41	0	16	25	51	*p *= 0.003*

* *p *< 0.05

### Relationship between Clinicopathological Characteristics and *EIF4G1 *Expression in Patients with NPC

The relationship between clinicopathological characteristics and *EIF4G1 *expression in patients with NPC is summarized in Table 1. We did not find any significant association of EIF4G1 expression with age, sex, smoking status, or distant metastasis (M classification) in 132 patients with NPC. However, we observed that the levels of EIF4G1 expression were closely correlated with the T classification (T1-T2 *vs*. T3-T4) (*P *= 0.039), lymph node involvement (N classification, N0-N1 *vs*. N2-N3) (*P *= 0.008), and clinical stage (I-II *vs*. III-IV) (*P *= 0.003) in NPC patients.

To investigate the prognostic value of *EIF4G1 *for NPC, we assessed the association between the levels of *EIF4G1 *expression and patients' survival time in 107 NPC cases with prognosis information available. We observed that the expression levels of *EIF4G1 *were significantly related to the overall survival of NPC patients. Patients with high levels of *EIF4G1 *expression had significantly shorter overall survival time than those with low levels of *EIF4G1 *expression (*P *= 0.019) (Figure 1C). To estimate the clinical significance of various prognostic factors that might influence the survival and tumor progression in NPC patients, univariate analyses were performed. As summarized in Table 2, the lymph node involvement (N classification) (*P *= 0.032), clinical stage (*P *= 0.023), and high expression of *EIF4G1 *(*P *= 0.014) were statistically significant risk factors affecting the overall survival of NPC patients. To determine the independent prognostic effects of these variables, multivariate analyses were performed using the Cox's proportional hazards model. Results showed that only high expression of *EIF4G1 *can independently predict the overall survival of NPC patients (*P *= 0.027) (Table 2). Taken together, our findings indicate that the levels of *EIF4G1 *expression could serve as an effective biomarker for the prognosis of NPC patients.

**Table 2:** Summary of univariate and multivariate Cox regression analysis of overall survival duration

	Univariate analysis			Multivariate analysis		
		
Parameter	*P*	HR	95%CI	*P*	HR	95%CI
Age						
> 45 vs. ≤ 45 years	0.497	1.252	0.655-2.391			
Gender						
Male vs. female	0.966	1.015	0.516-1.997			
Smoking						
No vs. Yes	0.239	0.628	0.290-1.362			
T classification						
T_1_-T_2 _vs. T_3_-T_4_	0.122	1.631	0.877-3.031			
N classification						
N_0_-N1 vs. N_2--_N_3_	**0.032**	2.034	1.062-3.898	0.455	1.393	0.584-3.323
M classification						
M_0 _vs. M_1_	0.492	1.662	0.390-7.073			
Clinical stage						
I-II vs. III-IV	**0.023**	2.465	1.132-5.368	0.360	1.629	0.572-4.638
EIF4G1 expression						
High expression vs. Low expression*	**0.014**	3.678	1.308-10.344	**0.027**	3.251	1.147-9.214

*High expression: medium and high expression; **Low expression: **negative and low expression;

### Reduced *EIF4G1 *Expression Suppressed the Proliferation, Cell Cycle Progression, Migration, and Invasion of NPC Cells *in vitro*

**Figure 2:**
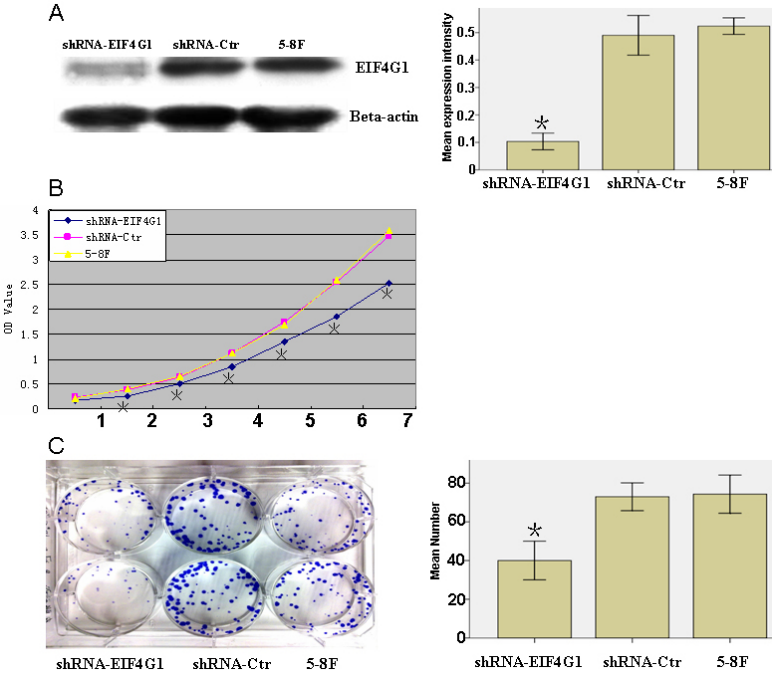
**Reduced expression of *EIF4G1 *inhibited cell proliferation**. **A**. Western blotting assay shows significantly decreased protein expression of *EIF4G1 *in shRNA-*EIF4G1 *cells comparing to shRNA-Ctr and the parental 5-8F cells. β-actin was used as the internal control. **B**. The cell growth of parental 5-8F cells and their stable derivatives, shRNA-Ctrl and shRNA-*EIF4G1*, were examined by MTT assay over a seven-day period. *P < 0.05, as compared to 5-8F and shRNA-Ctrl cells.

To study the biological function of *EIF4G1*, we used a lentiviral vector containing shRNA specifically targeting *EIF4G1 *to stably knock down the endogenous expression of *EIF4G1 *in 5-8F cells, an NPC cell line with high levels of endogenous *EIF4G1*. As shown in Figure 2A, comparing to the control (shRNA-Ctrl), cells transfected with shRNA-*EIF4G1 *had significantly decreased levels of *EIF4G1 *protein.

Subsequently, we examined the effect of decreased *EIF4G1 *expression on NPC cell growth *in vivo*. Using MTT assay, we found that the parental NPC 5-8F cells had a similar growth rate as shRNA-Ctrl cells over a seven-day period, while starting from day 2 the growth of shRNA-*EIF4G1 *cells was significantly slower than the former two cells (*P *< 0.05) (Figure 2B). Interestingly, the consistent result also appeared in the plate clone formation test. Both the parental 5-8F cells and the shRNA-Ctrl cells formed a similar number of colonies on plate over a two-week period [(73 ± 3.6) vs. (74.3 ± 4.93)]. In contrast, knocking down endogenous *EIF4G1 *could dramatically reduce the number of colonies (40 ± 5.0) (P < 0.05) (Figure 2C).

**Figure 3:**
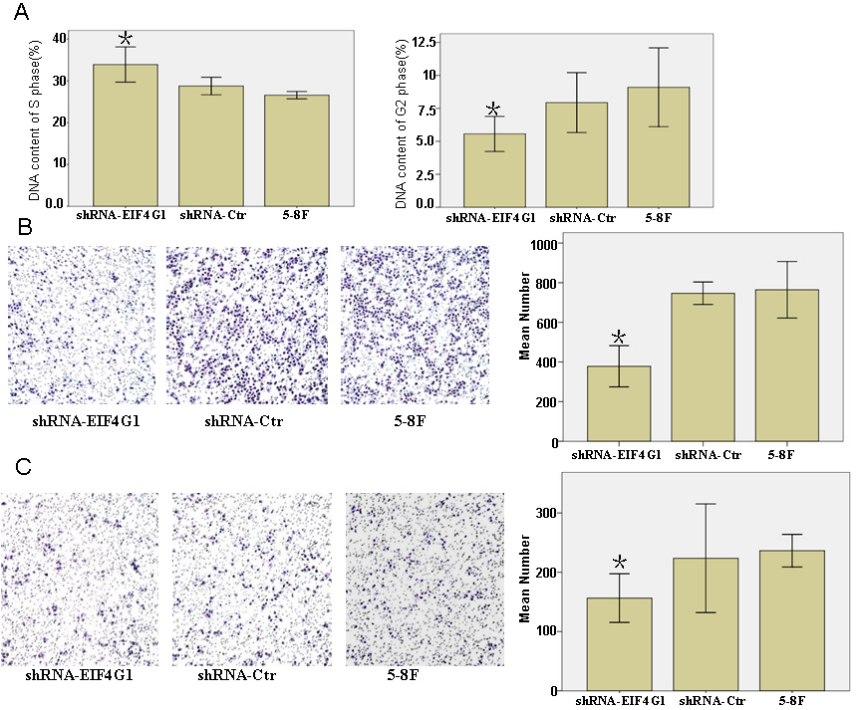
**Reduced *EIF4G1 *expression inhibited cell migration, invasion and cell cycle progression**. A. Cell cycle was determined by FACS Caliber Cytometry. Cell migration (B) and invasion (C) capabilities of parental 5-8F cells and their stable derivatives, shRNA-Ctrl and shRNA-*EIF4G1*, were examined using transwell assay. Data were presented as mean ± SD for three independent experiments. *P < 0.05, as compared to shRNA-Ctrl and 5-8F cells.

We also measured the alteration of cell cycle progression by *EIF4G1 *knock-down. Using flow cytometry analysis, we found that *EIF4G1*-deficient cells showed a significant increase in S phase proportion and a decrease in G2/M proportion compared to the shRNA-Ctrl and the parental 5-8F cells (*P *< 0.05) (Figure 3A).

Cell migration and invasion are integral steps for the process of tumor development and metastasis. When testing the abilities of 5-8F cells to migrate/invade through the 8-μm pores on the polycarbonate membrane with or without pre-coated matrigel, we found that the knock-down of endogenous *EIF4G1 *expression could significantly reduce cell migration and invasion as compared to the parental or shRNA-Ctrl cells (*P *< 0.05) (Figures 3B, C).

### Knock-down of *EIF4G1 *Inhibited *in vivo *Xenograft Tumor Growth

**Figure 4:**
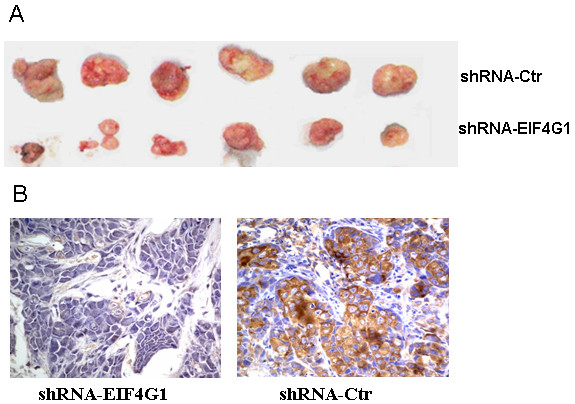
**Reduced *EIF4G1 *expression inhibited cell colony formation and xenograft tumor growth *in vivo***. **A**. The cell clone ability of parental 5-8F cells and their stable derivatives, shRNA-Ctrl and shRNA-*EIF4G1*, were examined by plate colony formation assay. *P < 0.05, as compared to 5-8F and shRNA-Ctrl cells. **B**. 5-8F cells containing shRNA-Ctrl or shRNA-*EIF4G1 *lentiviral vectors were injected into nude mice (n = 6 for each group). After 25 d, tumors were harvested and weighted. Data are presented as mean ± SD. *P < 0.05 as compared to shRNA-*EIF4G1 *cells. **C**. *EIF4G1 *protein expression was markedly decreased in xenograft tumors induced by shRNA-*EIF4G1 *cells comparing to that induced by shRNA-Ctrl cells.

In addition to examining the biological functions of *EIF4G1 in vitro*, we also assessed the *in vivo *function of *EIF4G1 *in a xenograft tumor transplantation model. After subcutaneously transplanting the cells containing shRNA-Ctrl or shRNA-*EIF4G1 *lentiviral vectors into nude mice, we monitored the tumor growth over a 25-day period. As shown in Figure 4A, by measuring the tumor weights, we found that shRNA-*EIF4G1 *cells gave rise to significantly smaller tumors than shRNA-Ctrl cells did (*P *< 0.05). Immunohistochemistry assay revealed that protein expression of *EIF4G1 *was significantly decreased in the tumors induced by shRNA-*EIF4G1 *cells compared to those induced by shRNA-Ctrl cells (Figure 4B), which was in consistent with the *in vitro *results in cells transfected with shRNA-*EIF4G1 *or the control vectors (Figure 2A).

### *EIF4G1 *Inhibited the Expression of *PDCD4 *in NPC cells

**Figure 5:**
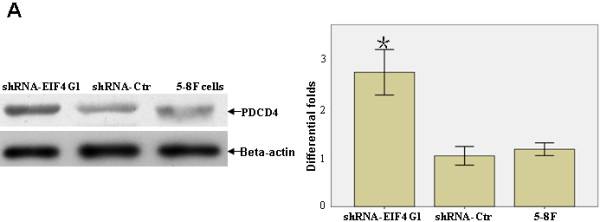
**Reduced *EIF4G1 *expression activated the expression of *PDCD4 *protein**. **A**. Protein expression of *PDCD4 *was increased in shRNA-*EIF4G1 *cells compared to shRNA-Ctrl cells and the parental 5-8F cells. Data were presented as mean ± SD. *P < 0.05.

The above results indicated that over-expression *EIF4G1 *may play an important role in promoting the development of NPC. We further examined the effect of *EIF4G1 *on the expression of *PDCD4*, a key tumor suppressor protein interacting with *EIF4G1 *and controlling tumor growth and invasion [19]. We speculated that reducing the levels of *EIF4G1 *activates the effects of *PDCD4*. To test this hypothesis, we measured the protein levels of *PDCD4 *in cells deficient of *EIF4G1*. Comparing to the parental 5-8F cells and cells containing the control vector, *EIF4G1*-deficient cells had increased levels of *PDCD4 *protein (Figure 5A). Our results suggest that *EIF4G1 *may be involved in the development of NPC by antagonizing the effect of *PDCD4*.

## Discussion

It is well known that the majority of NPC deaths result from tumor metastases rather than from primary tumors. However, the molecular mechanisms underlying the regulation of tumor cell invasion and metastasis of NPC remain incompletely understood. *EIF4G1*, a member of the translational initiation factor family, is recognized as the central organizing protein in the recruitment of mRNA during translational initiation. Gradually accumulated evidences indicate that increased *EIF4G1 *expression may play an oncogenic role and promote the process of tumorigenesis. For instance, over-expression of *EIF4G1 *has been found in inflammatory breast cancer (IBC) and promotes the formation of IBC tumor emboli by enhancing translation of IRES-containing *p120 *mRNA, which facilitated tumor cell survival [15]. In addition, frequent amplification of *EIF4G1 *on chromosome 3q27.1 and over-expression of *EI4G1 *mRNA was also displayed in lung cancer and hypopharyngeal cancer [13,14], which suggest the involvement of *EIF4G1 *in tumorigenesis [11,20]. Yet, it remains largely unknown about the role of *EIF4G1 *in the pathogenesis of NPC.

In the previous microarray study, we found that the levels of *EIF4G1 *mRNA in NPC were increased compared to noncancerous nasopharyngeal tissues [7]. In this report, we further presented the proof that *EIF4G1 *was over-expressed at both mRNA and protein levels in NPC tissues and cell lines. Furthermore, we found that *EIF4G1 *over-expression was significantly associated with T classification, lymph node involvement, and clinical stages of NPC patients. Our results also indicated that *EIF4G1 *protein expression was inversely correlated with patients' overall survival time. NPC patients with higher expression levels of *EIF4G1 *protein had shorter survival time, and the protein levels of *EIF4G1 *were an independent prognostic factor. These results suggested the clinical significance of *EIF4G1 *as a biomarker for NPC prognosis.

To understand the biological functions of *EIF4G1*, we employed the loss-of-function approach, that is, by knocking down the expression level of endogenous *EIF4G1*. To achieve this purpose, we chose to use NPC 5-8F cells, a cell line with high tumorigenic and metastatic ability. The 5-8F cells exhibit the highest expression levels of endogenous *EIF4G1 *in all the six NPC cell lines examined, and thus represent an ideal model for our study. Using this system, we identified the roles of *EIF4G1 *in promoting cell proliferation, cell cycle, migration, and invasion; we further found that *EIF4G1 *could enhance tumorigenesis *in vivo*. These results strongly suggest an oncogenic role of *EIF4G1 *in NPC development.

*EIF4G1 *may be used as a useful molecular marker for NPC and an indicator for tumor progression and prognosis. In combination with other biomarkers of NPC, *EIF4G1 *would be useful for novel therapeutic strategies. Assembly of the *EIF4E/EIF4G *complex plays a central role in the regulation of gene expression at the level of translation initiation. This complex is regulated by the *4EBP1*, a translational repressor that inhibits the function of eukaryotic translation initiation factor 4E (*EIF4E*), which compete with *EIF4G1 *for binding to *eIF4E *and have tumor-suppressor activity[21]. To pharmacologically mimic 4E-BP function Moerke et al developed the most potent small-molecule compound 4EGI-1 for disrupting *EIF4E/EIF4G *association, which inhibited cap-dependent translation but not initiation factor independent translation. While 4EGI-1 displaces *EIF4G *from *EIF4E*, it effectively enhances *4EBP1*, a translational repressor that inhibits the function of eukaryotic translation initiation factor 4E (*EIF-4E*), association with *EIF4E *both in vitro and in cells, and suppressed cell proliferation [22,23]. Additionally, *EIF4G1 *has been reported to implicate in another signal pathway. For example, *EIF4A/EIF4G *complex has been shown to be involved in the key regulation of translation initiation. Tumor suppressor *PDCD4 *[24,25] could compete with *EIF4A *for binding to *EIF4G1 *and thus inhibit the initialization of translation [26]. Although *PDCD4 *is known to be a key regulator that controls tumor growth and invasion [27], the role of *PDCD4 *in NPC progression remains unclear. Recently, in a microarray study, we observed a significant down-expression of *PDCD4 *in NPC samples [7]. We therefore speculated that *EIF4G1 *may play a role in NPC by inhibiting the functions of *PDCD4*. In the present study, we found that the reduction of endogenous *EIF4G1 *protein expression increased the expression levels of *PDCD4*. This result supports the potential involvement of *EIF4G1 *in regulating *PDCD4 *expression.

In summary, our results provided the first evidence that *EIF4G1 *may be involved in the development of NPC. In addition, we also demonstrated that *EIF4G1 *could serve as a biomarker for the prognosis of NPC. Further works are needed to investigate the mechanisms and pathways of NPC pathogenesis mediated by *EIF4G1*.

## Materials and methods

### Cell Culture and Sample Collection

NPC cell lines 5-8F, 6-10B, CNE2, HONE1, HNE1, and SUNE1 were maintained in RPMI 1640 culture medium supplemented with 10% newborn calf serum (NBCS) (PAA Laboratories, Austria). NP69, an immortalized human nasopharyngeal epithelial cell line, was grown in defined-KSFM medium supplemented with EGF (Invitrogen, Carlsbad, CA). All of the cell lines were incubated in a humidified chamber with 5% CO_2 _at 37°C. Seven primary NPC tissues, 5 noncancerous nasopharyngeal tissues, 132 paraffin-embedded NPC specimens (107 cases with prognosis information), and 53 noncancerous paraffin-embedded nasopharyngeal specimens were obtained from People's Hospital of Zhongshan City, China, at the time of diagnosis before any therapy. An informed consent was obtained from each patient. All freshly collected samples were immediately stored in liquid nitrogen.

### Real-time PCR

Real-time PCR was performed to measure the expression of *EIF4G1 *mRNA in NPC cell lines, tumor tissues, or normal nasopharyngeal tissues using SYBR Premix Ex Taq (Takara, Japan) as described previously [28]. The primers used were: forward 5'-TTGTGGATGATGGTGGCT-3' and reverse 5'-TTATCTGTGCTTTCTGTGGGT-3'. *GAPDH *was used as an internal control and the primers were: forward 5'-GCACCGTCAAGGCTGAGAAC-3' and reverse 5'-TGGTGAAGACGCCAGTGGA-3'. Each measurement was repeated three times.

### Immunohistochemistry

Paraffin sections (3 μm) from 132 NPC and 53 nasopharyngeal samples were deparaffinized in 100% xylene and re-hydrated in descending ethanol series according to standard protocols [7]. Heat-induced antigen retrieval was performed in 10 mM citrate buffer for 2 min at 100°C. The endogenous peroxidase activity and non-specific antigens were blocked with peroxidase blocking reagent containing 3% hydrogen peroxide and serum. The slides were incubated with rabbit anti-human *EIF4G1 *antibody (1:50) (Cell Signaling Technology, CA, USA) for 1 h at 37°C. After washing, the sections were incubated with biotin-labeled goat anti-rabbit IgG antibody for 10 min at room temperature followed by incubation with streptavidin-conjugated horseradish peroxidase (HRP) (Maixin Inc, China). The peroxidase reaction was developed with 3,3-diaminobenzidine chromogen solution in DAB buffer substrate. The slides were reviewed and scored independently by two pathologists blinded to the clinical parameters. The staining intensity was scored as described [29,30]. The extent of the staining, defined as the percentage of positive staining cells in relation to the whole field, was scored on a scale of 0 to 3 as 0: < 10%; 1: 10-25%; 2: 26-75%; and 3: ≥ 76%. The sum of the staining-intensity and staining-extent scores (0-6) was used as the final staining score for *EIF4G1*. For statistical analysis, a final staining score of 0, 1~2, 3~4, and 5~6 were respectively considered to be negative, low, medium, and high expression levels.

### Establishment of NPC 5-8F cell line with stably expressing shRNA-*EIF4G1*

The construction of lentiviral vector of shRNA targeting *EIF4G1 *and establishment of NPC 5-8F cell line stably expressing shRNA had been described previously [17,18,31]. In brief, we selected one sequence for targeting the *EIF4G1 *gene using the BLOCK-It RNAi Designer (Invitrogen, Carlsbad, CA). The preparation of lentiviral vectors expressing human *EIF4G1 *short hairpin RNA (shRNA) was performed using the BLOCK-It Lentiviral RNAi Expression System (Invitrogen, Carlsbad, CA), following the manufacturer's instruction and replication-incompetent lentivirus was produced by cotransfection of the pLenti6/*EIF4G1 *expression vector and ViraPower packaging mix (Invitrogen) containing an optimized mixture of three packaging plasmids: pLP1, pLP2, and pLP/VSVG into 293FT cells. NPC 5-8F cells were infected with lentiviral particles containing specific or negative control vectors and selected for stable integrants by culturing a complete medium containing blasticidin. After 15 days of selection, there were no viable cells in mock wells and discrete blasticidin resistance colonies. The total RNA of these cell clones was isolated, and the levels of *EIF4G1 *mRNA were measured using real-time PCR as described above.

### Western blot Analysis

Cells were lysed in RIPA Buffer (50 mM Tris-HCl pH 8.0, 1 mM EDTA pH 8.0, 5 mM DTT, 2% SDS), and protein concentration was determined using BCA assay (Beyotime Inc, China). Total protein (30 μg) was resolved using a 10% SDS-PAGE gel and transferred onto a polyvinylidene fluoride (PVDF) membrane. The primary antibodies used were rabbit polyclonal anti-*EIF4G1 *antibody(1:500), anti-*ACTB *antibody(1:400) (both from Santa Cruz Biotechnology, CA, USA) and *PDCD4*(1:500, Abcam Inc., Cambridge, MA). An HRP-conjugated anti-rabbit IgG antibody was used as the secondary antibody (Zhongshan Inc, China).

### Cell Proliferation and Cell Cycle Analyses

Cell proliferation was analyzed using MTT assay (Sigma, St. Louis, USA). Briefly, 1 × 10^3 ^cells were seeded into a 96-well plate with quadruplicate repeat for each condition. After 72 h of incubation, MTT reagent was added to each well and incubated for 4 h. The formazan crystals formed by viable cells were then solubilized in DMSO and measured at 490 nm for the absorbance (A) values. Each experiment was performed in triplicates.

To evaluate cell cycle distribution, cells were seeded on 10 cm-diameter plates in RPMI 1640 culture medium containing 10% NBCS. After 48 h of incubation, a total of 1 × 10^6 ^cells were harvested, rinsed with cold PBS, and fixed with 70% ice-cold ethanol for 48 h at 4°C. Fixed cells were rinsed with cold PBS followed by incubation with PBS containing 10 μg/mL propidium iodide and 0.5 mg/mL RNase A for 15 min at 37°C. The DNA content of labeled cells was acquired using FACS Caliber cytometry (BD Biosciences). Each experiment was performed in triplicates.

### *In vitro *Cell Migration and Invasion Assays

Cells growing in the log phase were treated with trypsin and re-suspended as single-cell solution. A total of 1 × 10^5 ^cells were seeded on a fibronectin-coated polycarbonate membrane insert in a transwell apparatus (Corning Inc., Corning, NY). In the lower chamber, 600 μl of RPMI 1640 with 10% NBCS was added as chemoattractant. After the cells were incubated for 12 h, the insert was washed with PBS, and cells on the top surface of the insert were removed by a cotton swab. Cells adhering to the lower surface were fixed with methanol, stained with Giemsa, and counted under a microscope in five predetermined fields (×200). For the Matrigel invasion assay, the procedure was similar with the cell migration assay, except that the transwell membrane was precoated with ECMatrix™ and the cells were incubated for 14 h. All assays were independently repeated at least three times.

### Colony Formation Assay

About 100 cells were added to each well of a 6-well culture plate, and each cell group contained 2 wells. After 2 weeks of incubation, cells were washed twice with PBS and stained with Giemsa solution. The number of colonies containing ≥ 50 cells was counted under a microscope. The colony formation efficiency was calculated as: efficiency = (number of colonies/number of cells inoculated) × 100%. Each experiment was performed in triplicates.

### Growth of Tumor Xenografts in Nude Mice

All animal studies were conducted in accordance with the principles and procedures outlined in the National Institutes of Health (NIH) Guide for the Care and Use of Animals under assurance number A3873-1. Nude mice of 4 to 6 weeks old (n = 6) were purchased from the Animal Center, Southern Medical University. A total of 1 × 10^6 ^cells growing in log phase were re-suspended in serum-free RPMI 1640 medium and injected subcutaneously into the nude mice. To minimize inter-individual variation, cells containing control vector and cells containing shRNA-*EIF4G1 *vector were injected symmetrically to the either flank of the same mice. After 25 d, mice were sacrificed and tumors isolated and weighted. *EIF4G1 *protein levels in the xenograft tumors were examined using immunohistochemistry assay.

### Statistical analysis

All data were analyzed for statistical significance using SPSS 13.0 software. The Mann-Whitney U test was applied to the analysis of relationship between *EIF4G1 *expression levels and clinicopathologic characteristics. Survival analysis was performed using Kaplan-Meier method (the log-rank test). Multivariate Cox proportional hazards method was used for analyzing the relationship between the variables and patient's survival time. One-way ANOVA was used to determine the differences between groups for all *in vitro *and *in vivo *analyses. A *P *value of less than 0.05 was considered statistically significant.

## Disclosure of Potential Conflict of interests

The authors declare that they have no competing interests.

## Authors' contributions

LT, ZL, and WF carried out the molecular and cell biology studies. WF and ZL participated in the design of the study and performed the statistical analysis, collected, analyzed, and interpreted data and wrote the manuscript. XL interpreted data. GX revised the manuscript. XH, QJ, YH and SX collected and analyzed data. WF, KY supervised all the work. All authors have read and approved the final manuscript.
